# Plant composition changes in a small-scale community have a large effect on the performance of an economically important grassland pest

**DOI:** 10.1186/s12898-019-0248-6

**Published:** 2019-09-04

**Authors:** Xinghu Qin, Huihui Wu, Xunbing Huang, T. Ryan Lock, Robert L. Kallenbach, Jingchuan Ma, Md. Panna Ali, Xiongbing Tu, Guangchun Cao, Guangjun Wang, Xiangqun Nong, Mark R. McNeill, Zehua Zhang

**Affiliations:** 1 State Key Laboratory for Biology of Plant Diseases and Pests, Institute of Plant Protection, Chinese Academy of Agricultural Sciences, Beijing, 100193 People’s Republic of China; 2 Present Address: Scottish Oceans Institute, Institiud Chuantan na h-Alba, School of Biology, University of St Andrews, East Sands, St Andrews, Scotland KY16 8LB UK; 3 Scientific Observation and Experimental Station of Pests in Xilingol Rangeland, Ministry of Agriculture, Xilinhot, 026000 People’s Republic of China; 4 Analysis Centre for Agricultural of Experiments of Tianjin Agriculture, Tianjin, 300384 People’s Republic of China; 5 Division of Plant Sciences, University of Missouri, 108 Waters Hall, Columbia, MO 65211 USA; 6 Entomology Division, Bangladesh Rice Research Institute (BRRI), Gazipur, 1701 Bangladesh; 7 Biocontrol & Biosecurity, AgResearch, Private Bag 4749, Christchurch, 8140 New Zealand

**Keywords:** Plant composition, Grasshopper plague, Plant stoichiometric traits, Grassland conservation

## Abstract

**Background:**

The grasshopper *Oedaleus asiaticus* Bey-Bienko (Acrididae: Oedipodinae) is a dominant and economically important pest that is widely distributed across the Mongolian plateau. This herbivore pest causes major damage to the grassland of the Inner Mongolian steppe in China. The population dynamics of herbivore pests is affected by grassland management practices (e.g., mowing and heavy livestock grazing) that alter plant community structures and stoichiometric characteristics. For example, *O. asiaticus* outbreak is closely associated with plant preference changes caused by nitrogen loss from heavy livestock grazing. However, the manner by which small-scale variation in vegetation affects grasshopper performance and promotes outbreak is poorly characterized. To address this question, we investigated the relationship between small-scale (1 m^2^) vegetation variability and measures of *O. asiaticus* performance associated with plant stoichiometric characteristics.

**Results:**

We found that food preferences of *O. asiaticus* varied significantly, but maintained a specific dietary structure for different plant compositions. Notably, small-scale changes in plant community composition significantly affected grasshopper food preference and body size. Partial least-square modeling indicated that plant proportion and biomass affected grasshopper body size and density. We found that this effect differed between sexes. Specifically, female body mass positively correlated with the proportion of *Stipa krylovii* grass, whereas male mass positively correlated with the proportion of *Artemisia frigida* grass. Further analyses indicated that grasshopper performance is closely associated with plant stoichiometric traits that might be responsible for the pest’s plague.

**Conclusions:**

This study provides valuable information for managing grasshoppers using rational grassland management practices.

## Background

The grasshopper is an important grassland inhabitant that directly impacts ecological and economic factors in agro-ecosystems [1]. *Oedaleus asiaticus* Bey-Bienko (Acrididae: Oedipodinae) is a dominant and economically important grasshopper species that causes major damage to China’s grassland [2, 3]. This species is also a bio-indicator of habitat deterioration in the typical steppe regions [4]. Many studies have found that *O. asiaticus* population density negatively correlates with plant biomass [5–8] and their feeding habits change significantly depending on livestock grazing pressures and plant community composition [9]. Therefore, changes in the composition of plant communities may have complicated consequences on grasshopper biology and food preferences. For stakeholders to assess and improve the value of grasslands as demand for food increases, they must develop a comprehensive understanding of all biotic processes that occur in these agro-ecosystems [10].

The diet and distribution of grasshoppers are largely determined by their food preferences and food availability. Studies continue to report that grassland management practices, such as mowing and heavy livestock grazing, cause major changes in herbivores’ diet composition and preferences, as well as changes in plant physiology and nutrition [9, 11–15]. Thus, understanding grasshopper food preferences, and the factors that determine their feeding patterns, is necessary for developing grassland protection and management strategies.

*Oedaleus asiaticus* is a rarely studied herbivore, but it was recently reported that its outbreak is closely associated with an altered food preference caused by plant nitrogen changes in response to heavy livestock grazing [16, 17]. Measures of *O. asiaticus* performance, including food preference and body size, are closely related to grasshopper migration and outbreak [17, 18]. However, the relationship between grasshopper performance and plant composition and stoichiometric characteristics is complex and poorly understood.

Diet quality is a major extrinsic influence on herbivores [19] because nutrients are a major determinant of herbivore performance, including growth and reproduction [20, 21]. However, the mechanisms determining diet composition are poorly understood. It is known that food preferences not only depend on the biomolecule content, but also the presence of certain mineral elements [21, 22]. Also, an organism’s diet must ensure growth and reproduction, in addition to meeting metabolic demands [23]. Several hypotheses have been proposed to explain diet composition, including the nutrient complementation hypothesis (NCH) [21, 24, 25] and toxin dilution hypothesis (TDH) [26]. There has also been progress with the identification of some ingestive and post-ingestive regulatory mechanisms in herbivore insects that are necessary for nutritional balance [27, 28].

Interestingly, plant stoichiometric traits, including leaf carbon content (LCC) and the carbon: nitrogen (C:N) ratio, are known to affect the palatability of plants to *O. asiaticus* [16]. Another stoichiometric trait, the nitrogen:phosphorus (N:P) ratio of an organism’s body, is closely linked to species’ performance, measured by development rate, body size, survival [21, 22]. Indeed, the N:P ratio of a species usually has a narrow range of variation relative to C:N and C:P ratios [21, 22]. Furthermore, P is an indispensable element and the relative ratio of P could determine grasshopper survival. Increased dietary P content has been observed to increase growth rate and improve survival in insects [28–31]. However, other research has found either no effect or negative effects of dietary P intake in insects [24, 25]. Although P is widely considered an important growth factor for diverse biota [28, 29, 31], few studies have explored the effect of P content, C:P ratio, and N:P ratio on grasshopper performance.

At the community level, plant composition relates closely to grassland management practices [32]. In moderation, anthropogenic management practices such as grazing positively impact biodiversity in Inner Mongolian grassland [33]. Typically, diverse plant types of shortgrass and mixed prairie grass, comprise an Inner Mongolian steppe and highly uneven livestock grazing occurs. Large-scale geographic plant composition can affect insect herbivores, which are generally more abundant where their food plants are highly available [34, 35]. Overall, the large-scale factors that influence grasshopper growth, development, survival, and reproduction are known, and include temperature, soil, microstructure, and others [7, 16, 36]. However, the impact of small-scale vegetation variation on *O*. *asiaticus* remains unclear. Though recent studies indicated that plant distribution pattern and resource imbalance impact locust phase [37, 38], these studies unintentionally neglected the difference between plant proportion and plant biomass composition in contributing to the performance of locust. Therefore, this study sought to understand how plant composition (plant proportion and plant biomass composition) at the small-scale community level affects *O. asiaticus* performance. Additionally, we investigated whether plant stoichiometric traits such as plant N, C, and P contents and ratios, correlate with grasshopper performance.

## Results

### Host plant food preference

The host preference test showed that *O. asiaticus* consumed a large proportion of *S. krylovii*, *C. squarrosa*, and *L. chinensis* grasses, indicating a preference for these plants (Additional file 1: Figure S1). Of these three preferred species, the relative feeding frequency was highest for *S. krylovii* and lower for *C. squarrosa* and *L. chinensis* (Additional file 1: Figure S1). There was limited feeding on *A. frigida*, *C. microphylla*, and *C. ammannii*, and no feeding on *K. prostrata* and *N. pectinata*. Notably, all of these non-preferred plants are forbs.

### No-choice field experiments

*Oedaleus asiaticus* consumed significantly different amounts of the three preferred plant species (*F* = 182.52; df = 2, 17; *P* < 0.0001). Intake of *S. krylovii* was highest, followed by *L. chinensis*, and then *A. frigida* (Additional file 1: Figure S2a). Food consumption results were consistent with survival rates. Survival rates did not differ significantly between *S. krylovii* and *L. chinensis* consumption, nor between *L. chinensis* and *A. frigida*; however, survival rates were significantly different between *S. krylovii* and *A. frigida* (Additional file 1: Figure S2b; *F* = 4.97; df = 3, 14; *P* = 0.027).

### Small-scale plant community composition changes affect grasshopper food consumption

**Fig. 1:**
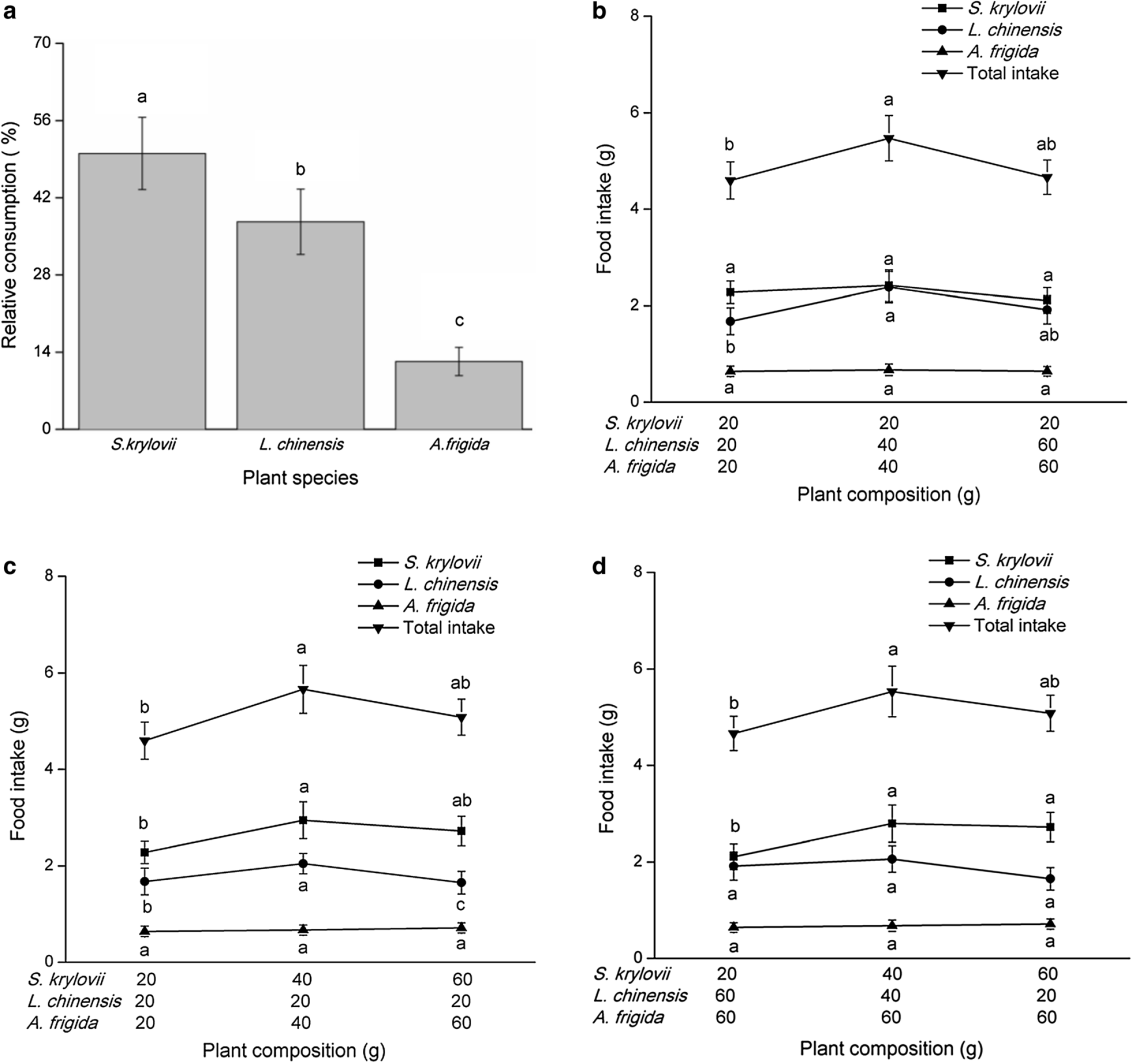
Mean dietary intake (± SEM) of *Oedaleus asiaticus* (from fourth-instar to adult) under manipulated ratios of the three plant species. **a** Relative consumption of the three plants (from fourth-instar to adult). **b** Constant *S. krylovii* biomass, altered *L*. *chinensis* and *A*. *frigida* biomass. **c** Constant *L. chinensis* biomass, altered biomass for the other two plant species. **d** Constant *A. frigida* biomass, altered biomass of the other two plant species. The horizontal axis of **b**–**d** indicates a plant composition that consisted of *S*. *krylovii*, *L. chinensis*, and *A. frigida*. Significant differences marked by different lowercase letters, based on Tukey’s HSD at *P* < 0.05

Results showed that changes in the proportions of plant species, or plant composition, did not change feeding patterns, although the proportional intake of each of the three plants was significantly different (Fig. 1a; *N* = 108; *F* = 470.31; df = 2107; *P* < 0.0001). Grasshopper diets were composed of 50.0 ± 6.56% *S. krylovii*, 37.6 ± 5.93% *L. chinensis*, and 12.3 ± 2.56% *A. frigida*. The three situations for the consumption test are summarized in Fig. 1b–d.

Grasshopper total food intake increased significantly as the total plant biomass increased to an intermediate level, but food intake slightly decreased when plant biomass was very high (*P* < 0.001, N = 25; Fig. 1b–d). *S. krylovii* intake (*N* = 25; *F* = 0.15; df = 2, 24; *P* = 0.8600) was relatively constant in situation b; however, when *S. krylovii* increased from low to intermediate biomass, there was a large increase in food intake (situation c, d). However, *S. krylovii* intake did not increase when plant biomass increased from intermediate to high (Fig. 1b–d). Similarly, intake of *L. chinensis* significantly increased (situation b: *N* = 25; *F* = 4.60; df = 2, 24; *P* = 0.0214 and situation c, *N* = 25; *F* = 3.82; df = 2, 24; *P* = 0.0375) when plants increased from low to intermediate biomass (Fig. 1b, c). Interestingly, *L. chinensis* intake remained stable when the total plant biomass was constant, but *L. chinensis* biomass decreased (situation d). For the least-preferred plant species, *A. frigida*, there was no significant difference in total absolute consumption between any of the treatments (Fig. 1b–d).

### Plant community composition affects grasshopper performance

**Fig. 2:**
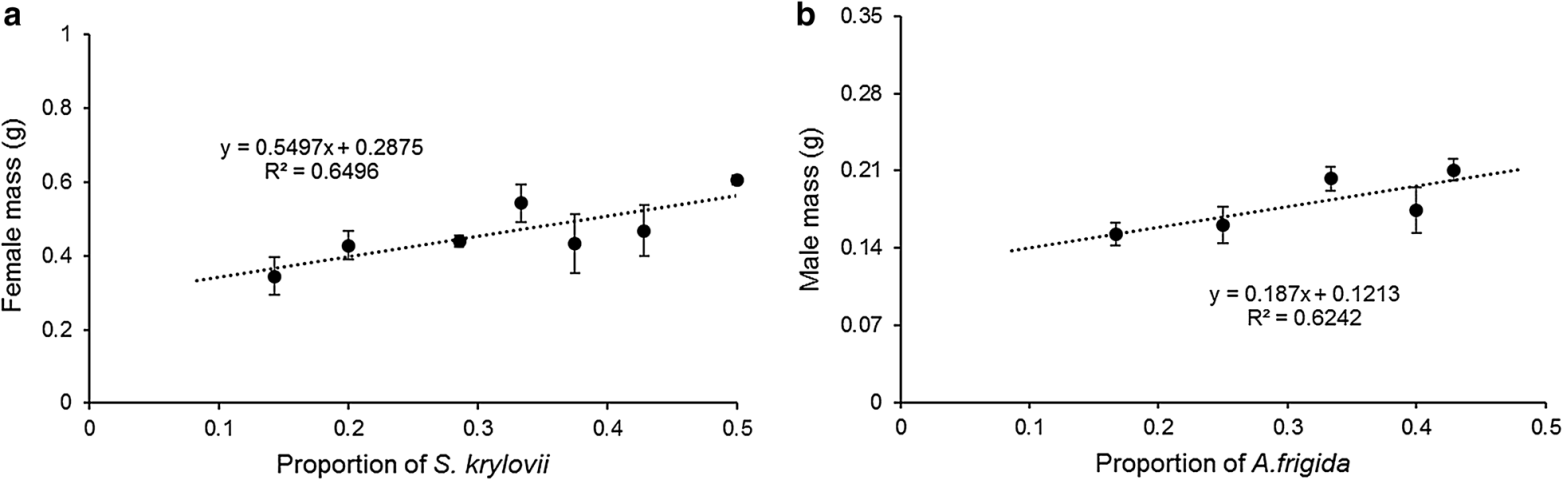
**a** The linear relationship between female mass and the dietary proportion of *S. krylovii* (*N* = 32; *r* = 0.39086; *P* = 0.0270). **b** The linear relationship between male mass and dietary proportion of *A. frigida* (*N* = 30; *r* = 0.50606; *P* = 0.0335). Significant differences marked by different lowercase letters, based on Tukey’s HSD at *P* < 0.05. Error bars indicate standard error

Field-diet experiments showed that survival rates (time series) were not significantly different for the different plant compositions offered in small-scale cages (1 m × 1 m × 1 m) (Additional file 1: Table S3). Multivariate variance analysis indicated that the total above-ground plant biomass significantly impacted overall grasshopper performance (Table 1). However, different plants had different effects on specific grasshopper performance parameters. For example, *A. frigida* biomass significantly influenced the first principal component of the grasshopper performance variables, and *S. krylovii* and *L. chinensis* significantly influenced the second principal component of grasshopper performance variables (Table 1). Further analyses indicated that female grasshopper mass significantly positively correlated with the proportion of the preferred plant *S. krylovii* (Table 2; Fig. 2a). Conversely, the proportion of the non-preferred plant, *A. frigida*, significantly positively correlated with male grasshopper mass and length (Table 2; Fig. 2b). Additionally, we found interacting effects of the relative proportions of *S. krylovii*, *L. chinensis*, and *A. frigida*, on male grasshopper mass. Together, these findings suggest that sexually dimorphic grasshoppers have different responses to the plant community changes.

**Table 1:** Multivariate analysis of the effect of the plant biomass (three level of plant biomass) on overall performance of grasshoppers

	Df	Pillai	Approx. F	den Df	Pr (> F)	Significance
Total model						
Sk	1	0.366	7.21	25	0.00338	**
Lc	1	0.459	10.60	25	0.00046	***
Af	1	0.560	15.92	25	3.48E−05	***
Residuals	26					

	Df	Sum Sq	Mean Sq	F value	Pr (> F)	Significance
PC1						
Sk	1	3.34	3.34	2.679	0.114	
Lc	1	3.54	3.54	2.8409	0.104	
Af	1	34.56	34.56	27.7	1.68E−05	***
Residuals	26	32.43	1.25			
PC2						
Sk	1	7.65	7.65	10.02	0.00393	**
Lc	1	16.23	16.23	21.25	9.41E−05	***
Af	1	1.42	1.42	1.85	0.185	
Residuals	26	19.86	0.764			

PC1, PC2 indicate the first and second principal components of grasshopper performance variables

Sk: *S. krylovii*; Lc: *L. chinensis*; Af: *A. frigida*

Statistical significance: ‘***’, 0.001; ‘**’, 0.01; ‘*’, 0.05; ‘.’, 0.1; ‘ ’, 1

**Table 2:** Analysis of variance of effect of different plant proportions on grasshopper performance variables

Grasshopper performance	Community parameters	Df	Sum Sq	F value	Pr (> F)	Significance
Male mass	Total plant biomass	1	0.00081	0.119	0.734	
	PSk	1	0.00531	0.779	0.387	
	PLc	1	0.0323	3.838	0.060	.
	PAf	1	0.0663	9.723	0.005	**
	PSk: PLc	1	0.00951	1.395	0.250	
	PSk: PAf	1	0.00508	0.745	0.397	
	PAf: PLc	1	0.0007	0.102	0.752	
	PSk: PAf: PLc	1	0.0299	4.38	0.048	*
	Residuals	22	0.15002			
Male length	Total plant biomass	1	0.00003	0.045	0.833	
	PSk	1	0.00038	0.578	0.455	
	PLc	1	0.00130	1.863	0.183	
	PAf	1	0.00298	4.53	0.045	*
	PSk: PLc	1	0.00051	0.78	0.387	
	PSk: PAf	1	0.00092	1.403	0.249	
	PAf: PLc	1	0.00112	1.707	0.205	
	PSk: PAf: PLc	1	0.00047	0.719	0.406	
	Residuals	22	0.014446			
Female mass	Total plant biomass	1	0.0403	2.605	0.121	
	PSk	1	0.0985	6.363	0.019	*
	PLc	1	0.0123	0.796	0.382	
	PAf	1	0.0640	4.106	0.0523	
	PSk: PLc	1	0.000	0.000	0.999	
	PSk: PAf	1	0.000	0.000	0.985	
	PAf: PLc	1	0.0086	0.558	0.463	
	PSk: PAf: PLc	1	0.0002	0.014	0.906	
	Residuals	22	0.341			
Female length	Total plant biomass	1	0.00264	1.647	0.213	
	PSk	1	0.00002	0.015	0.904	
	PLc	1	0.00155	1.123	0.298	
	PAf	1	0.00131	0.817	0.376	
	PSk: PLc	1	0.00041	0.256	0.618	
	PSk: PAf	1	0.00017	0.103	0.751	
	PAf: PLc	1	0.00006	0.035	0.854	
	PSk: PAf: PLc	1	0.00048	0.298	0.591	
	Residuals	22	0.03523			
Preference for Sk	Total plant biomass	1	0.04	1.19	0.287	
	PSk	1	8.151	241.24	2.43E − 13	***
	PLc	1	0.104	3.071	0.0936	.
	PAf	1	3.08	11.84	0.00184	**
	PSk: PLc	1	0.267	7.9	0.010185	*
	PSk: PAf	1	0.691	20.45	0.000169	***
	PLc: PAf	1	0.001	0.015	0.9036	
	PSk: PLc: PAf	1	0.369	10.919	0.0032	**
	Residuals	22	0.743			
Preference for Lc	Total plant biomass	1	0.16	7.026	0.0146	*
	PSk	1	0.763	33.42	8.14E−06	***
	PLc	1	3.245	142.15	4.5E−11	***
	PAf	1	1.153	7.464	0.011	*
	PSk: PLc	1	0.301	13.20	0.001	**
	PSk: PAf	1	0.45	19.72	0.0002	***
	PLc: PAf	1	0.012	0.505	0.485	
	PSk: PLc: PAf	1	0.042	1.828	0.190	
	Residuals	22	0.502			
Preference for Af	Total plant biomass	1	0	0	0.999739	
	PSk	1	0.138	13.80	0.00121	**
	PLc	1	0.191	19.21	0.00024	***
	PAf	1	0.321	35.51	2.04E−06	***
	PSk: PLc	1	0.020	1.964	0.175	
	PSk: PAf	1	0.00106	0.107	0.747	
	PLc: PAf	1	0.00218	0.219	0.645	
	PSk: PLc: PAf	1	0.00281	0.282	0.601	
	Residuals	22	0.21926			

PSk, PLc, PAf represent the proportion of *S. krylovii*, *L. chinensis*, and *A. frigida* in the community

Sk: *S. krylovii*; Lc: *L. chinensis*; Af: *A. frigida*

Statistical significance: ‘***’, 0.001; ‘**’, 0.01; ‘*’, 0.05; ‘.’, 0.1; ‘ ’, 1

We found that changing the proportion of the three plants affected grasshopper food preferences (Table 2). Changes in the proportion of either *S. krylovii* or *A. frigida* significantly influenced the preference for the other two plants (Table 2). All the plant pairs except *S. krylovii* and *A. frigida* had significant interaction effects on the preference for *S. krylovii* and *L. chinensis*, whereas *A. frigida* had no interaction effects (Table 2). However, the proportion of *L. chinensis* did not influence the grasshoppers’ preference for *S. krylovii*.

In our experiments, changes in the plant community structure (community dissimilarity) significantly influenced grasshopper food preferences for all three plants (Table 3). Notably, only male grasshopper mass dissimilarity was significantly affected by plant community structure (Table 3). Typical of sexual dimorphism, we observed that *O. asiaticus* body length and mass were significant different between sexes (male length vs. female length, N = 32, *P* = 4.59e−20; male mass vs. female mass, N = 32, *P* = 6.67e−25), and females were significantly larger and heavier than males.

**Table 3:** Effect of plant structure on grasshopper trait dissimilarity

Grasshopper traits	Factors	Df	Sum Sq	R^2^	F value	Pr (> F)	Significance
Male length	Community dissimilarity	7	0.88	0.31	1.39	0.26	
	Residual	22	1.99	0.69			
	Total	29	2.88	1			
Male mass	Community dissimilarity	7	0.85	0.44	2.46	0.0497	*
	Residual	22	1.08	0.56			
	Total	29	1.93	1.00			
Female length	Community dissimilarity	7	0.18	0.13	0.45	0.869	
	Residual	22	1.24	0.87			
	Total	29	1.42	1.00			
Female mass	Community dissimilarity	7	0.18	0.13	0.45	0.863	
	Residual	22	1.24	0.87			
	Total	29	1.42	1.00			
Preference for Sk	Community dissimilarity	7	2.04	0.93	40.68	0.0001	***
	Residual	22	0.16	0.07			
	Total	29	2.20	1.00			
Preference for Lc	Community dissimilarity	7	1.83	0.91	31.12	0.0001	***
	Residual	22	0.18	0.09			
	Total	29	2.01	1.00			
Preference for Af	Community dissimilarity	7	0.88	0.62	5.08	0.0027	**
	Residual	22	0.54	0.38			
	Total	29	1.42	1.00			

Significance analysis based on 9999 permutations

Sk: *S. krylovii*; Lc: *L. chinensis*; Af: *A. frigida*

Statistical significance: ‘***’, 0.001; ‘**’, 0.01; ‘*’, 0.05; ‘.’, 0.1; ‘ ’, 1

### Plant community composition affects grasshopper population density through food availability and quality, and biomass

Though greater plant biomass likely increases the amount of preferred food, we observed a weak negative effect of plant biomass composition (all three plants) on food availability and quality (Additional file 1: Table S4). However, the plant community biomass composition negatively affected grasshopper population density (*P* = 0.00179, Additional file 1: Table S4). Conversely, though plant biomass showed direct positive effects on grasshopper body size, the total effect was not significant (*P* = 0.1487) because of plant biomass indirect negative effects on grasshopper body size (Additional file 1: Table S4, Figure S4). Food availability and quality affects grasshopper population density (*P* = 0.00974) but not body size (*P* = 0.0887). Although grasshopper population density had a direct positive effect on grasshopper body size (coefficient is 0.3918), the effect is not significant in our study (*P* = 0.5701; Additional file 1: Table S4, Figure S4).

### Grasshopper performance is associated with plant stoichiometric characteristics

**Fig. 3:**
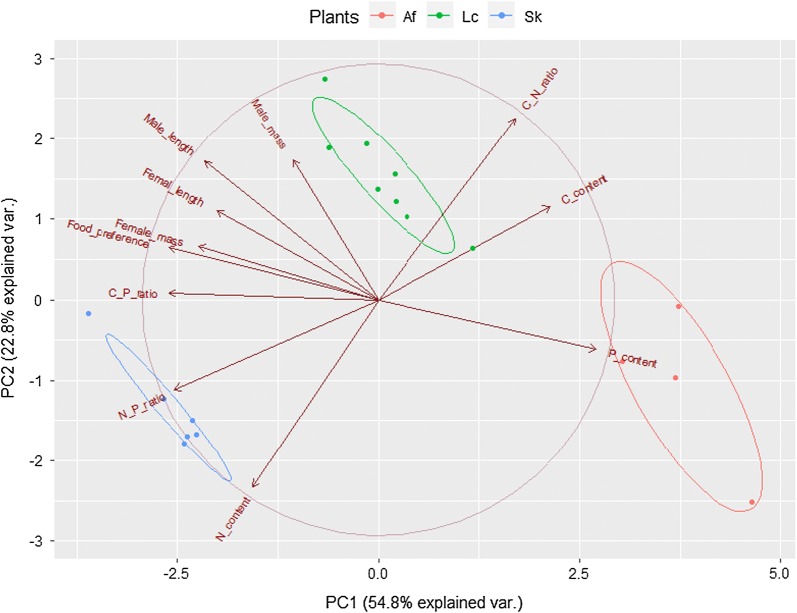
Ordination classification of relationships between measures of grasshopper performance and plant stoichiometric characteristics. The arrows indicate the grasshopper performance measures (male mass, male length, female mass, female length) and plant stoichiometric characteristics (C, N, P contents, C:N ratio, C:P ratio, and N:P ratio). X-axis and Y-axis represent the first and second principal components of these variables. Ellipses indicate plant types corresponding to *S. krylovii* (Sk, blue), *L. chinensis* (Lc, green), and *A. frigida* (Af, red)

Principal component analysis indicated that grasshopper performance parameters, including male mass, male length, female length, female mass, and grasshopper food preference clustered together closely, and these variables had strong positive correlations with C:P and N:P ratios (Fig. 3). However, these variables negatively correlated with P content (Fig. 3). We also observed that the plant with the lowest P content, *S. krylovii*, was highly consumed by grasshoppers, and the plant with the highest P content, *A. frigida* was lowly consumed by grasshoppers during development from the third instar to the adult stage. The above results suggest that C:P ratio, N:P ratios and P content may contribute to the differences in grasshopper performance variables, especially food preference.

**Fig. 4:**
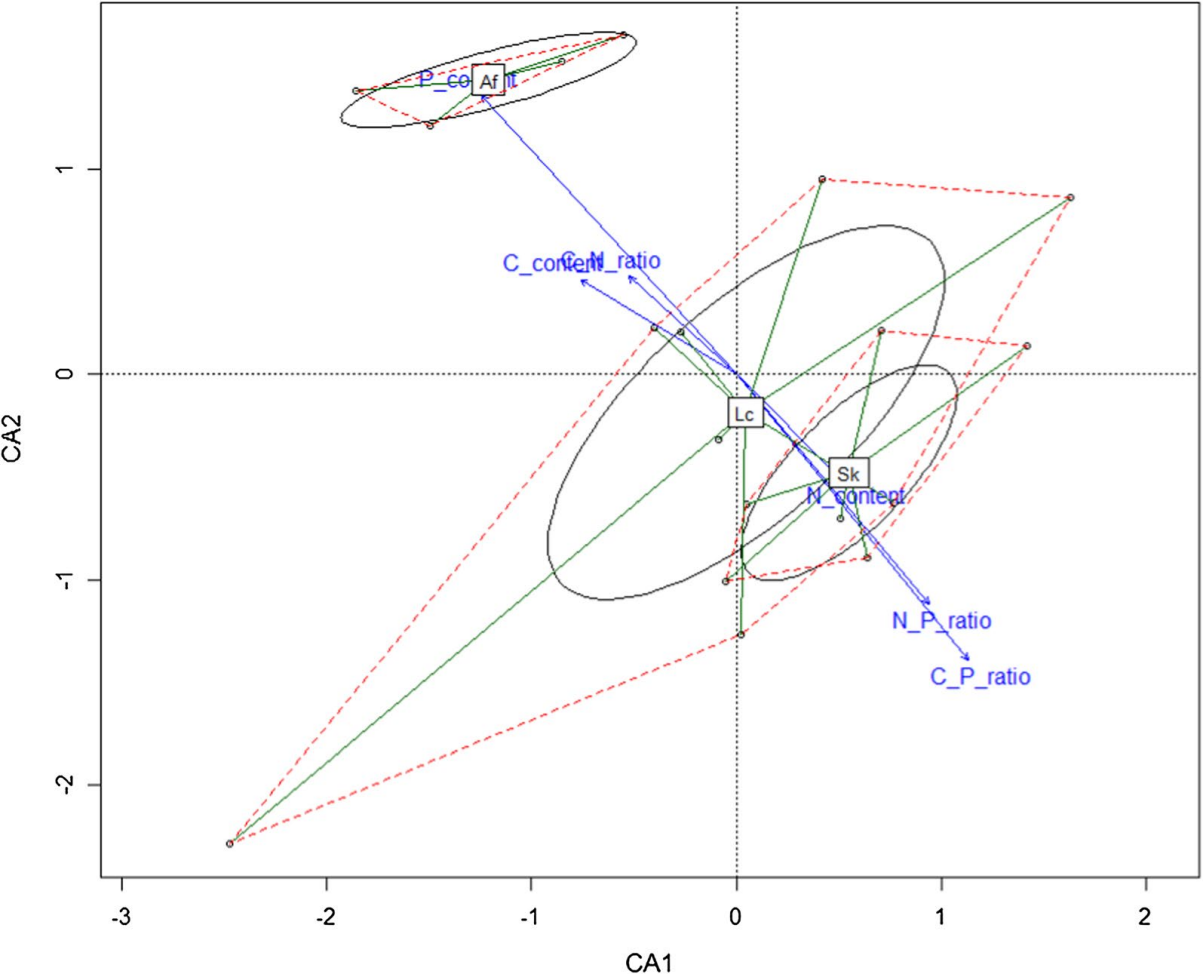
Association between grasshopper performance and plant stoichiometric characteristics. X-axis and Y-axis indicate the first two ordination scores of grasshopper performance variables. The convex hulls (red) enclose centroids of grasshopper performance measures obtained from grasshoppers fed with the corresponding plants, *S. krylovii* (Sk), *L. chinensis* (Lc), and *A. frigida* (Af). The ellipses enclose confidence areas (95%) of grasshopper performance measures. The blue arrows indicate the plant stoichiometric characteristics for three plants

We identified relationships between variables in different scenarios using canonical correspondence analysis. We found that grasshopper performance clustered into three groups that corresponded to the three plants (Fig. 4, *Sk*, *S. krylovii*; *Lc*, *L. chinensis*; *Af*, *A. frigida*). All variables are presented as a linear correspondence, and grasshopper performance linearly correlated with the corresponding food preference level (Fig. 4). Three stoichiometric characteristics: P content, C:P ratio, and N:P ratio (Table 4), significantly correlated with grasshopper performance variables (Fig. 4). Low-performing grasshoppers clustered into the non-preferred plant group (*Af*, *A. frigida*), in contrast to high-performing grasshoppers that clustered into the preferred plant groups (*Sk*, *S. krylovii*; *Lc*, *L. chinensis*). High performance variables tended to group with high C:P and N:P ratios, whereas low performance variables tended to group with higher P content. These findings suggest that higher C:P and N:P ratios contributed to high grasshopper performance and higher P content contributed to low grasshopper performance, in the context of these three plants. We found that the approximate grasshopper C:N:P dietary ratio is 249.8:18.8:1.0, based on consumption of the three plant species and their C, N and P content. Overall, this finding indicates that *O. asiaticus* maintained relatively high dietary C:N and C:P ratios, but low dietary P content (0.18%).

**Table 4:** Goodness of fit of the association between grasshopper performance and plant stoichiometric characteristics from constrained correspondence analysis

Stoichiometric traits	CA1	CA2	r2	Pr (> r)	Significance
N content	0.701	− 0.713	0.117	0.368	
P content	− 0.674	0.739	0.761	0.000	***
C content	− 0.854	0.521	0.178	0.205	
C, N ratio	− 0.739	0.674	0.113	0.378	
C, P ratio	0.630	− 0.776	0.722	0.000	***
N, P ratio	0.642	− 0.766	0.479	0.005	**

Significance analysis based on 9999 permutations

Statistical significance: ‘***’, 0.001; ‘**’, 0.01; ‘*’, 0.05; ‘.’, 0.1; ‘ ’, 1

## Discussion

In this study, we sought to understand how plant stoichiometric traits and ratios in small-scale plant communities influence the performance of the grasshopper *O. asiaticus*, an economically significant pest. We determined food preferences using eight plant species in laboratory choice experiments, and further investigated grasshopper diet structure by feeding grasshoppers three different preferred plant species in a field choice experiment where plant composition was manipulated in small-scale (1 m × 1 m × 1 m) communities. Grasshoppers showed different preference for several plant species reflected by selective indices (*S. krylovii*, > *L*. *chinensis*, > *A*. *frigida*) and survival rates (*S. krylovii*, > *L*. *chinensis*, > *A*. *frigida*). However, despite altering the proportion of each plant’s biomass in the community, the grasshoppers regulated their intake of each plant species and did not simply feed on each plant according to its relative abundance. Plant community composition changes significantly influenced grasshopper performance, including grasshopper body size, food preference and population density. We also found that plant P content and C:P and N:P ratios correlated with grasshopper performance variables, suggesting that plant stoichiometric characteristics play important roles in the maintenance of *O. asiaticus* dynamics. Additionally, male and female grasshoppers performed differently within plant community structures, with female body mass positively correlated with the proportion of *S. krylovii* grass, whereas male mass positively correlated with the proportion of a non-preferred plant, *A. frigida*, suggesting niche divergence between sexes.

### *O. asiaticus* diet preference and structure

Regardless of changes in plant community composition, grasshoppers tended to eat the same amount of *S. krylovii*, *L*. *chinensis*, and *A*. *frigida*. This observation suggests that *O. asiaticus* has a highly specific diet. In addition, female grasshoppers achieved a greater mass with a higher proportion of *S. krylovii*. This finding is consistent with the idea that increasing the proportion of a plant would benefit the growth of herbivores that prefer feeding on that plant.

*Oedaleus asiaticus* preference for certain plants in the presence of alternative foods was likely due to constraints on food quality or to the need to maintain a nutrient balance. A diet composed of several types of plants likely indicates that different foods are complementary nutritional resources for *O. asiaticus*. Previous studies have shown that *O. asiaticus* refused to eat *A. frigida* [39] and could not complete their life cycle when fed exclusively on *A. frigida.* However, we observed that *O. asiaticus* included *A. frigida* in their diet. In addition, increased *A. frigida* consumption correlated with increased *A. frigida* biomass, though not significantly.

Huang et al. [40] reported that when available plants were all grasses, consumption of each plant increased as their respective biomass increased. However, we note that all the plants in that study were preferred grasses (*S. krylovii*, *C. squarrosa* and *L*. *chinensis*) that had a constant total biomass (90 g), which is an unrealistic scenario outside of an experiment. Unlike that study, our plant communities consisted of three typical plants belonging to two families. We identified higher, medium, and lower preference levels for our three plants, amongst all the plants we tested in the grassland. In addition, we did not find food replaceable among the three plants; rather, we found the special role of non-preferred plant in contributing to the sex-specific traits.

*Oedaleus asiaticus* is a sexually dimorphic grasshopper with divergent morphology differences. We found that male body mass positively correlated with the proportion of *A. frigida*, indicating that *A. frigida* promotes male grasshopper mass. Sexually dimorphic traits in each sex were positively associated with the proportion of two plants (*S. krylovii*, *A. frigida*) with converse food preference by grasshoppers. This implies that food trophic structure and niche divergence between sexes play functional roles in supporting phenotypic diversity (within species diversity), and allow grasshoppers to adapt to changing grassland communities.

One drawback in previous studies of *O. asiaticus* is that none documented the interaction of gender with other variables in the field. Our data indicate this grasshopper is sexually dimorphic, and sex-specific phenotypes are sensitive to different factors. Therefore, we suggest that researchers test relevant biological characteristics of this grasshopper by sex to better understand it’s ecological and evolutionary processes.

### Grasshopper performance is associated with food availability and nutrient content

#### Plant proportion and biomass affect grasshopper performance

The relationship between plant community composition and grasshopper food preference and body size indicate that the *O. asiaticus* diet is influenced by mechanisms regulating plant composition. Nevertheless, why *O. asiaticus* chooses to consume a constant amount of *S*. *krylovii*, *L. chinensis* and *A. frigida* was still not well understood. Food preference and consumption depend on plant traits, such as plant structure, water content, toughness, or element content [27, 41–43]. Changes in plant composition, due to mowing or livestock activity, might change plant physical traits such as plant structure and toughness [44–46]. However, these changes might not be the determinant factor in promoting grasshopper outbreak in the grassland.

Earlier studies showed that food quality, physical contact, stimuli and population density triggers the aggregation of locusts [37, 38, 43, 47–50]. Although we set the population density to one that did not lead to density-dependent polymorphism and cannibalism [8, 51, 52], partial least-square modelling showed that plant biomass negatively affected grasshopper rearing density (*R* = − 0.7837, *P* = 0.00179, Additional file 1: Table S3). Therefore, the imposed density may impact the grasshopper performance. Our results showed that grasshopper population density affected grasshopper body size, but the total effect became masked by plant biomass, food availability, and quality (Additional file 1: Table S4, Figure S4). An earlier study indicated that high population densities caused grasshoppers to exhibit some morphological traits predicted to benefit migration (gregarious phase), but did not induce gregarious behaviour such as flight. This likely resulted as the migratory phase of *O. asiaticus* is partially triggered by high population densities, but also existing ecological conditions blocked full expression of such traits [51].

We determined from partial least-square modelling that plant biomass, together with *food availability and quality* had strong direct positive effects and weak indirect negative impacts on grasshopper body size. However, these factors negatively affected the grasshopper density (Additional file 1: Table S4; Figure S4). Previous studies about this grasshopper indicated that plant ground cover and biomass negatively influenced grasshopper population density [5, 7]. However, the mechanism underlying this phenomenon remains unclear because of difficulty in quantifying the effects of plant composition in the studies. One possible explanation is that both the quality and distribution of food resources at the local scale enhanced the probability of individual locusts making physical contact with each other and inducing phase change [38, 53]. Consistent with this explanation, our results reveal that both food availability and quality and plant composition (proportion and biomass) at a small scale impact grasshopper phenotype and density. In conjunction with a recent study [8], our study provides insight into mechanisms by which plant community structure regulates grasshopper population dynamics through the trade-off between body size and survival.

#### Plant stoichiometry affects grasshopper performance

Our study further investigated the role of plant nutrient traits on grasshopper performance. These traits correlated to plant composition changes often brought on by livestock grazing, mowing or other anthropogenic activities.

Recent studies of the relationship between heavy livestock grazing, plant composition, soil stoichiometry, and herbivore dietary stoichiometry indicate that plant composition changes might influence grasshopper performance by changing the nutrient composition of the grasshopper diet [17, 28, 54]. Thus, we hypothesized that nutrient elements required for a balanced diet might drive *O. asiaticus* performance, especially feeding preference. Ecologists have focused mostly on N, which is an estimate of plant protein content, and more recently, on P, with respect to feeding behavior [28, 54]. Although P has long been considered an important factor in the growth of many organisms, such as bacteria, algae, and zooplankton, the effect of P on insect herbivores is relatively unknown [54]. However, there is strong evidence that P content and C:P and N:P ratios are linked to grasshopper performance, especially feeding preference [28].

Indeed, contrary to expectations from P-limitation paradigms, the plant with the lowest P content, *S. krylovii*, was highly consumed by grasshoppers during development from the third instar to the adult stage. CCA analysis identified the relationship between plant stoichiometric traits and grasshopper performance, especially food preference (Table 3; Fig. 4). Our results suggest that high C:P and N:P ratios improve grasshopper performance, whereas high P content worsened grasshopper performance. However, it is important to note that plants with higher C:P and N:P ratios and lower P content would not necessarily always be favorable food for *O. asiaticus*, because continual high N content and low P content food would also unbalance stoichiometry for grasshoppers. Nutritional balance may be as or more important than the absolute amount of any one nutrient [21, 55].

The maintenance of a stable diet in an environment with changing food composition indicates a positive feedback between forage quality and food availability, encouraging grasshoppers to maintain nutrient balance. Herbivores and their food plants can have interacting effects on each other’s C:N:P ratio through various direct and indirect mechanisms [20]. Increased grasshopper density reduces the plant standing biomass and dramatically changes the C:N:P stoichiometry of both plants and litterfall [54, 56], possibly due to the selective pressure of grasshopper selective feeding.

Based on our findings on grasshopper consumption of the three plant species in this study and their C, N, and P content, we found that the *O. asiaticus* dietary ratio of C:N:P is 249.8:18.8:1.0. This ratio indicates that *O. asiaticus* not only prefers plants with relative low N, as shown in earlier studies, but also plants with very low P content [16]. Though grasshoppers were subjected to changing availability of plants and nutrients, they ate similar amounts of *S. krylovii*, *L*. *chinensis*, and *A*. *frigida*, suggesting that grasshoppers were balancing C, N, and P in their diets. Previous research has found similar evidence for dietary nutrient balancing in insects. For example, the American grasshopper *Schistocerca americana* Drury self-selects to ensure approximately 0.6% P content [28].

The diet of specialist herbivores is well explained by specific behavioral, morphological, and biochemical adaptations between the herbivore species and its host plants [57]. Both food availability and nutrient content in host plants influence the food preferences of *O. asiaticus*. Furthermore, grasshoppers had a higher preference for a target plant species when it was relatively scarce. It is known that grasshoppers independently select food when resources are more abundant, coexisting with other herbivores with different feeding patterns in the steppe, such as the grasshopper *Dasyhippus barbipes* Auktor preferring *L. chinensis* to *S. krylovii* [58].

Research indicates that feeding patterns are consistent with the regulation of food control and intake by direct metabolic feedback [59]. The specific and dynamic dietary structure we observed suggest that *O. asiaticus* maintains nutritional balance by changing its feeding behavior to accommodate food availability. Changes in the biomass of any one of the three plants altered the *SI* values of the other plants. This finding indicated that grasshoppers selected alternative resources to meet own requirements when faced with changing food availability. If grasshoppers regulate their diets to maintain nutrient balance, the grasshoppers can be considered to be behaviorally adjusting their food selection to obtain target quantities of nutrients [28]. These insights provide us with a clearer understanding of how and why grasshoppers might attempt to maintain a relatively constant dietary ratio of key host plants.

### Implications for grasshopper control and grassland conservation

**Fig. 5:**
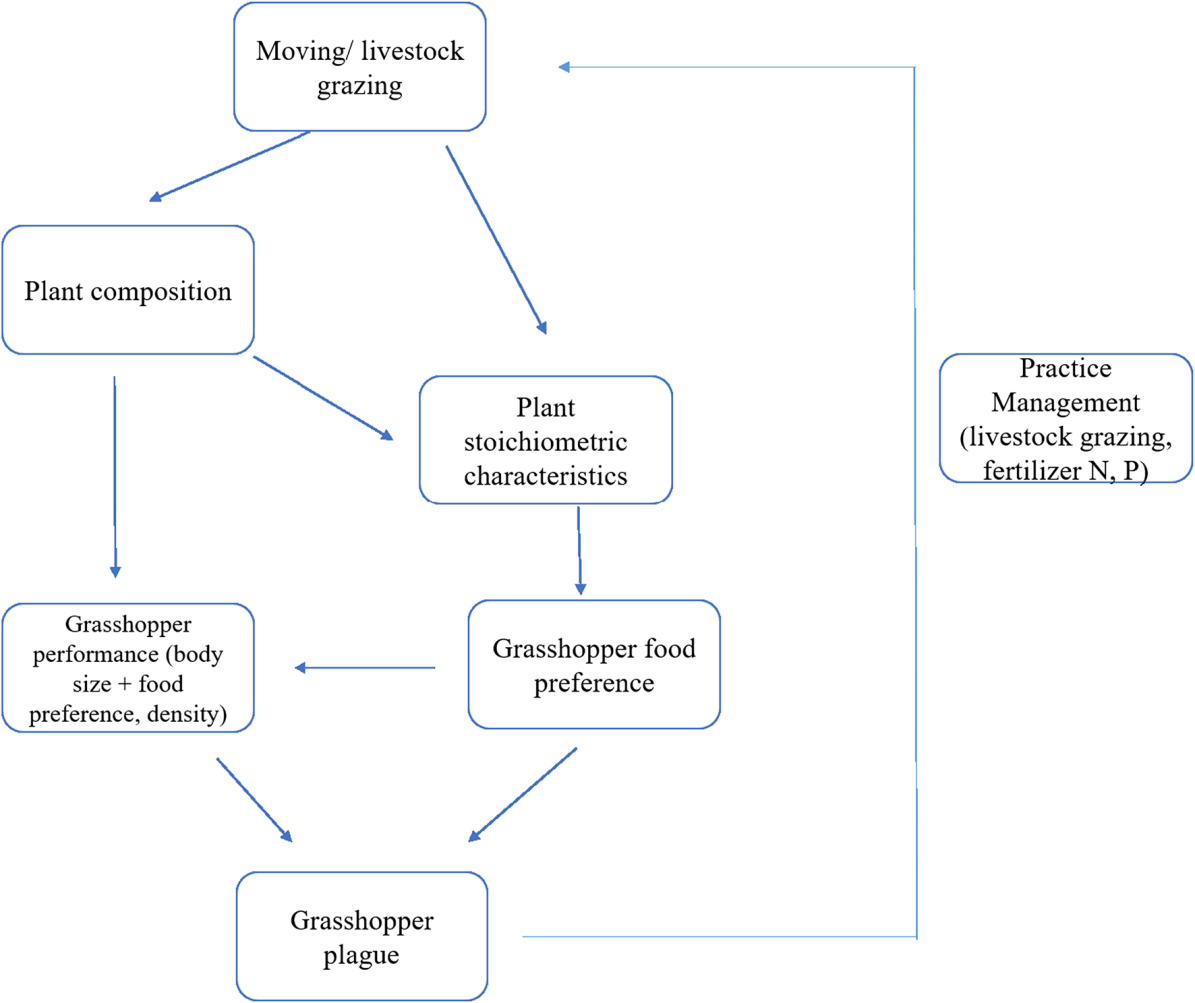
Implications of grassland management—grasshopper plague circulation

Although community composition did not affect grasshopper mortality, it impacted grasshopper body size, even in small-scale communities. Plant biomass on the other hand affected grasshopper population density rather than body size. Grasshopper body size is directly related to its migratory phase [8, 18], and density is the trigger of locust aggregation [60]. Both enable long flights and damage-causing plague. Therefore, finding approaches that change or regulate body size and population density provides solutions to sustainable pest management. For example, rational crop management like rotational grazing (intermittent grazing and deferment) [61, 62], or periodic grass mowing [63, 64], helps avoid locust outbreaks by impacting grasshopper performance. Additionally, we show that plant stoichiometric characteristics correlate with grasshopper performance, and that plant P may be related to *O. asiaticus* food preference. Since long-term livestock grazing can change the stoichiometric traits of some grasshopper preferred plants [65], grassland grazing and fertilization management practices [16, 17, 66], become tools for grasshopper pest control (Fig. 5).

Grassland management practices, including mowing and grazing, may influence grassland pest prevalence. Heavy livestock grazing or mowing can change plant community composition, degrade grassland, and cause loss of plant elemental nutrients [16, 65], ultimately promoting grasshopper outbreak. Additionally, sexually dimorphic *O. asiaticus* had distinguishable sex-specific benefits from different dietary plants, and plant community composition significantly influenced grasshopper sex-specific traits such as body size. These findings suggest that resource managers employ rational grassland practices to regulate grasshopper dynamics and control outbreaks. In summary, our study provides additional support for the idea that herbivore responses to their plant communities can be used to influence herbivore performance [67, 68], and provides new insights for improving agricultural management strategies to prevent economically damaging pest outbreaks.

## Conclusions

We studied the performance of *O. asiaticus* under different small-scale plant community compositions.

1. We found that when the grasshopper *O. asiaticus* had access to a range of plant species, grasshoppers had different degrees of food preferences, but maintained a species-specific dietary structure.
2. Small-scale plant composition changes had large effects on the performance of sexually dimorphic grasshoppers, which selectively fed on mixed plant communities in proportions that were favorable to different sexes.
3. Two characteristics of plant communities, plant proportion and biomass, affect grasshopper body size and density in different ways and regulate grasshopper performance through the trade-off of grasshopper phenotype and survival.
4. The C, N, P content, C:P, N:P ratios, and food availability of specific plants are potential influences on *O. asiaticus* performance.

This study provides insight into specific mechanisms by which differences in small-scale vegetation structure affect the interactions among individuals and cause changes in their biology that impact their status as a pest. The results give us valuable information to develop guidelines for grassland management and pest control. Importantly, future studies must clarify the relationship between plant stoichiometric characteristics and long-term grazing, to determine whether long-term livestock grazing changes P content in plants. An early step would be to explore the impact of the P on grasshopper performance by increasing P fertilization of plants.

## Methods

### Field site

Field experiments were conducted at the Scientific Observation and Experimental Station of Pests in Xilingol Rangeland, Ministry of Agriculture, Xilin Gol League, Inner Mongolia, China, (43°57′10.7″N 116°00′35.4″E). Laboratory host plant preference tests (E1) were performed in 2012. No-choice field experiments were performed in 2013 (E2). Field-diet experiments, including those examining the effect of small-scale plant composition changes on grasshopper performance parameters (E3), and plant chemical analyses (E4) were performed in 2014. Each year, we collected second-instar *Oedaleus asiaticus* using sweep nets within a fenced meadow near the station and subsequently reared grasshoppers in large insect-rearing cages (2 m × 1.5 m × 2 m) as a grasshopper pool. Based on previous research, we set grasshopper density below 25 individuals/m^2^ to avoid their aggregation and phase change [18, 50]. Thus, none of the grasshoppers in these experiments cannibalized each other.

### Host plant food preference test

To clarify the feeding habits of *O. asiaticus*, we offered the following eight plant species to individual grasshoppers for simultaneous choice tests under laboratory conditions: *Stipa krylovii* Roshev., *Leymus chinensis* (Trin.) Tzvel., *Cleistogenes squarrosa* (Trin.) Keng (all Poaceae), *Artemisia frigida* Willd., *Neopallasia pectinata* (Pall.) Poljak. (Asteraceae), *Caragana microphylla* Lam (Fabaceae), *Convolvulus ammannii* (Convolvulaceae), and *Kochia prostrata* (Chenopodiaceae). These are the most common plant species in the Xinli Gol grassland, a typical Eurasian steppe grassland, and provide habitats for many native species (e.g., mammals, birds and insects) as well as high-quality forage for domesticated livestock.

For the plant choice bioassays, we collected adult grasshoppers from the rearing cages and then placed them in plastic crates (30 cm × 20 cm × 15 cm) in the laboratory. We tested 10 individuals (sex ratio 1:1) in each of the five groups, with five repetitions of each group. On the first day, the grasshoppers were put into plastic crates and starved for 24 h prior to the start of the experiment. We cut eight single fresh plants of each species from the nearby meadow, with stems being removed, weighed the plants, and then provided 2 g of each plant species to the grasshoppers after 24 h. Each plant species was inserted into water-filled petri dishes through individual slits on the petri dish lids. Every 2 days, dead grasshoppers and old leaves were replaced with live grasshoppers and fresh leaves. We recorded the number of feeding grasshoppers (*X*_*i*_) on each plant (*i*) and the number of foraging grasshoppers (*F*) hourly 10 times per day (8:00–18:00), and calculated the relative feeding frequency (RFN) accordingly. The consumed part of the supplied leaf was quantified (in mm^2^) using graph paper and paper templates used to reconstruct the original leaf size, based on a previously described method [16]. The preference level (*PL*) for each plant species was determined by consumption area according to the following scale: 0: not consumed (< 0.5% of the plant eaten); 1: limited consumption (0.5–30% of the plant eaten); 2: high consumption (> 30% of the plant eaten). Each experiment was repeated three times.

### No-choice field experiments

We performed no-choice experiments in clean gauze covered cages (1 m × 1 m × 1 m) in a sandy field where all vegetation had been removed. The results of the preference test described above showed that *O. asiaticus* tended to be a grass (*S. Krylovii*, *L. chinensis* and *C. squarrosa*) specialist that avoided forbs (*A. frigida*, *N. pectinata*, *C. microphylla*, *C. ammannii*, and *K. prostrata*). We used a subset of different preferred grasses and a forb, specifically *S. krylovii*, *L. chinensis* (grasses), and *A. frigida* (forb), for further investigation. We collected plants from the grassland close to the field and placed 20 g of each single plant species into plastic containers (14 cm × 20 cm) filled with distilled water. The containers were buried so that the top of the containers were flush with the ground surface. We placed 20 individual third-instar grasshoppers from the grasshopper pool (sex ratio 1:1) into each cage. The numbers of surviving grasshoppers in each treatment were recorded every 3 days until grasshoppers reached adulthood. After the grasshoppers reached adulthood, we conducted a plant consumption test. During the consumption test, we weighed an additional five bundles of each plant species before adding plants to the cages without grasshoppers. After weighing, plants were dried for 48 h and weighed again to estimate original dry mass (*O*) and the water content of the plant material. The remaining portions of uneaten leaves (*U*) were collected after 48 h, dried for 24 h at 80 °C, then weighed to determine the plant dry mass consumed. Plant consumption (*C*) was calculated by subtracting the dry mass of the uneaten leaves (*U*) from the dry mass of the original (*O*). We calculated the dry plant: wet plant ratio from a control group of an additional five leaf bundles for each plant species that were treated equivalently but without grasshoppers. Every 2 days, we quantified the amount of plant material consumed by the grasshoppers and replaced old leaves with new leaves of the same species. The consumption test lasted 2 weeks. We repeated the experiment five times so that the results of each 48-h foliage replacement provided three data sets for analysis.

### Effect of small-scale plant composition change on grasshopper performance

*Stipa krylovii*, *L. chinensis*, and *A. frigida* grassland are the most common components of a typical steppe in Xilingol. We used these three plant species to investigate grasshopper feeding patterns under small-scale field conditions (1 m^2^). According to our annual plant investigation, the average above ground plant biomass of a single plant ranges from 20 to 60 g/m^2^ in the steppe (http://nmg.cern.ac.cn/meta/detail/GA02). To replicate changing grassland composition, we performed the experiment using an orthogonal design assigning three factors to plant species (*S. krylovii*, *L. chinensis*, *A. frigida*) and three levels of plant community biomass (20, 40, and 60 g) (Additional file 1: Tables S1, S2). The nine scenarios, with the three plant species offered at different relative abundances, were repeated three times. Each plant species was placed in a container filled with water with a lid that allowed the stems of the plants to be inserted into the water but prevented the grasshoppers from drowning. Containers were then placed into holes so that the lids were flush with the ground surface.

We selected third-instar grasshoppers from the grasshopper pool to form groups of 20 individuals (sex ratio 1:1), which were then transferred to gauze cages (1 m × 1 m × 1 m) located at the field site. Grasshoppers were held in cages without food for 24 h before starting the experiment. We quantified food consumption every 2 days, which was when the plants were replaced. Every instar was measured three times over the duration of the experiment (from third instar to adulthood) with each treatment replicated three times. We recorded the numbers of survivors in each treatment every 3 days until the grasshoppers reached adulthood. This experiment lasted 30 days. Food consumption (*C*) was calculated by subtracting uneaten dry mass (*U*) from the original dry mass (*O*), as described above (2.3). We weighed all the plants using an analytical balance (Mettler/ML104, 0.0001 g).

We measured seven grasshopper performance parameters, including male body length, male body mass, female body length, female body mass, grasshopper trait diversity, and grasshopper food preference for each plant (*S. krylovii*, *L. chinensis*, *A. frigida*). All these variables are closely associated with grasshopper phase transition and plague [50]. Thus, they are informative metrics for grasshopper performance. We recorded grasshopper body length and mass from the fourth instar until the adult stage under different plant community compositions. Five males and five females were randomly selected from each cage and their body lengths (from head to tail) were measured with digital calipers. Body mass was measured using a precision electronic balance (Mettler/ML104, 0.0001 g). We evaluated food preference using the selectivity index (*SI*), which is defined as the ratio of the fraction of a given food type in the feeders’ diets to the fraction of the same food in the community [69]. In addition to plant composition, we also recorded the community structure among scenarios which was measured by Bray–Curtis dissimilarity [70] to estimate the community structure change.

### Plant chemical analyses

We determined the total C content of *S. krylovii*, *L. chinensis*, and *A. frigida* using the potassium dichromate heating method [71] and total N content using micro Kjeldahl distillation as described by Yoshida et al. [72]. The total P was analyzed colorimetrically with the ammonium molybdate method [73]. For all these plant content analyses, we collected the entire aboveground biomass of each plant in a 1-m^2^ quadrant and separated plants into leaf and stem material. We combined leaves (with sheaths and stems removed) for a given species for each sample, then dried and ground leaves into a fine powder using a grinding machine (Yongkang Hardware Co, BL-500A). The powder was then passed through a 100-μm mesh screen. Thus, each sample was a composite of all the leaf blades from a given species collected from a 1-m^2^ quadrant. Three samples from different quadrat were measured.

### Statistical analysis

We calculated plant consumption (*C*_*i*_, g/d/individual) by the different instars using the formula:

1 $$C_{i} = \, \left( {O_{i} {-}U_{i} } \right)/(T*S)$$

where *O*_*i*_ is the dry mass of the intact leaves from an estimate of the original dry mass for a given plant species (*i*) and *U*_*i*_ is the dry mass of the remaining leaves for a different plant species (*i*) after being eaten. *S* is the number of grasshoppers surviving at the end of each period (*T*). We calculated the relative feeding frequency using the formula:

2 $$RFN_{i} = \, X_{i} /F$$

where *X*_*i*_ is the number of foraging grasshoppers in the *i* plant and *F* is the total number of foraging grasshoppers.

The dietary selectivity index (*SI*) was calculated using the formula [74]:

3 $$SI_{i} = \left( {D_{i} - P_{i} } \right)/\left( {D_{i} - P_{i} - 2D_{i} P_{i} } \right)$$

where *D*_*i*_ is the percentage of each plant (*i*) in the grasshoppers’ diet and *P*_*i*_ is the percentage of that plant’s biomass in the community [9, 69, 74, 75]. We calculated mean food intake and preference of each plants for grasshopper at each instar, and used grasshopper adult performance variables to perform principal component analysis (PCA), canonical correspondence analysis (CCA) and regression analyses.

We conducted multivariate analysis of variance to test the effect of plant composition on overall grasshopper performance and on each performance variable. Overall grasshopper performance was determined by obtaining the first principle component scores for all grasshopper performance parameters (male body length, male body mass, female body length, female body mass, and grasshopper food preference for each plant). A multiple variance test was conducted on the variance of three different preference levels for each of the three plants on overall grasshopper performance using *manova* in R [76]. We independently tested variances for main and interaction effects of each plant proportion on grasshopper performance parameters using ANOVA (*aov*) in R. In addition, we tested the effect of changing plant community structure on grasshopper trait diversity. We used Bray–Curtis dissimilarity [70] and Gower’s dissimilarity [77] to measure community structure and grasshopper trait diversity (dissimilarity), respectively. The variance of community structure for grasshopper trait dissimilarity at the community level was analyzed using the *vegan* package [78].

Grasshopper length and mass were logarithmically transformed, and a *P* < 0.05 was the threshold for statistical significance in all tests.

### The association between grasshopper performance and plant stoichiometric characteristics

We used constrained correspondence analysis (CCA, also called canonical correspondence analysis) to fit the association between plant stoichiometric characteristics and ordinations of grasshopper performance. CCA enables the projection of grasshopper performance ordinations (points) onto plant stoichiometric characteristics (vectors) to maximize the correlation between them. Briefly, we obtained ordination scores of grasshopper performance variables using *cca* function with *vegan* software (R package). We then fitted plant stoichiometric characteristics onto grasshopper performance ordinations using *envfit* function (*vegan* package). Statistical significance for goodness of fit was obtained based on 9999 permutations. We then visualized an association plot following five steps for the *ordispider*, *ordiellipse*, *ordihull* functions in the *vegan* software package.

### Partial least-square path modelling on grasshopper performance

Though we kept grasshopper population density constant, changing plant composition led to changes in plant biomass and grasshopper food availability and quality. These outcomes indirectly modified population density of grasshoppers. Food availability and quality, and grasshopper density are two important factors that trigger aggregation of grasshopper [17, 37, 38, 47]. To reveal whether population density affected grasshopper performance in our experiment, we performed partial least-square path modelling on grasshopper performance parameters. The latent variables in our model are *plant biomass composition* (four variables: biomass of each plant and the total biomass), *food availability and quality* (three variables: the availability and quality index of each plant) and *grasshopper density* (two variables: fifth-instar density and adult density). The dependent variables are grasshopper body size parameters (four variables: male and female body size). *Food availability and quality* represents the proportion of the plants and their preferences by grasshoppers. Therefore, in our structure model, the parameter “*availability and quality index*” (matrix) can be obtained from the multiplication of the proportion of each plant in the given community and the selectivity index of each plant for grasshoppers. The presentation of our model path is shown in Additional file 1: Figure S3.

The implementation of partial least square modelling was conducted in R using *plspm* package [79], 999 bootstrapping was used to test the significance of the model.

## Supplementary information

**Additional file 1: Table S1.** The factors that are used for orthogonal design. **Table S2.** Orthogonal design and their factors. **Table S3.** Variance analysis of the orthogonal test for mortalities of *O. asiaticus* from third instar to adults within manipulated plant community compositions. **Table S4.** Partial least-square path modelling of the effects (coefficient) of plant biomass, food availability and quality, and grasshopper population density on grasshopper body size. **Figure S1.** Feeding frequency and preference level of eight plants *PL*s for *S. krylovii*, *L. chinensis*, *C. squarrosa* are 2 other plants are defined as 0 PL: preference level; 0: not consumed (< 0.5% of the plant eaten); 1: limited consumption with 0.5–30% of the plant eaten; 2: high consumption with > 30% of the plant eaten Sample size *N* = 50. **Figure S2.** Mean consumption (g/day/individual ± SEM) (a) and survival rate ± SEM (b) of *O. asiaticus* feeding on three plant species as measured under laboratory conditions The horizontal axis indicates the plant species. Sample size, *N* = 60 The values of the bar chart are mean ± SEM. Error bar indicates the standard error. Those marked by different lowercase letters are significantly different based on Tukey’s HSD at *P* *<* 0 05. **Figure S3.** The partial least-square modelling path on grasshopper performance. **Figure S4.** The effect of plant biomass composition on grasshopper body size from partial least-square modelling (Red arrows indicate negative effects, blue arrows indicate positive effects).

## Data Availability

All the datasets on grasshoppers and plants at the study site that used and analyzed for this study are included in the article and its Additional file. Raw dataset are available from the corresponding author on reasonable request.

## Abbreviations

*SI*: dietary selectivity index
*O*_*i*_: the dry mass of the intact leaves from an estimate of the original dry mass for a given plant species (*i*)
*U*_*i*_: the dry mass of the remaining leaves for the different plant species (*i*) after being eaten
*S*: is the number of grasshoppers surviving at the end of each period (*T*)
*RFN*_*i*_: the relative feeding frequency
*X*_*i*_: is the number of foraging grasshoppers in the *i* plant
*F*: is the total number of foraging grasshoppers
*D*: is the percentage of each species (*i*) in the diet
*P*_*i*_: is the percentage of the same species biomass in the community
Sk: *S. krylovii*
Lc: *L. chinensis*
Af: *A. frigida*
