# Supplementary material for: Plant composition changes in a small-scale community have a large effect on the performance of an economically important grassland pest
Source: BMC Ecol. 2019 Sep 4;19:32. doi: 10.1186/s12898-019-0248-6 (PMC6727414; doi:10.1186/s12898-019-0248-6)
Supplement: Supplementary file 1 — Additional file 1: Table S1. The factors that are used for orthogonal design. Table S2. Orthogonal design and their factors. Table S3. Variance analysis of the orthogonal test for mortalities of O. asiaticus from third instar to adults within manipulated plant community compositions. Table S4. Partial least-square path modelling of the effects (coefficient) of plant biomass, food availability and quality, and grasshopper population density on grasshopper body size. Figure S1. Feeding frequency and preference level of eight plants PLs for S. krylovii, L. chinensis, C. squarrosa are 2 other plants are defined as 0 PL: preference level; 0: not consumed (< 0.5% of the plant eaten); 1: limited consumption with 0.5–30% of the plant eaten; 2: high consumption with > 30% of the plant eaten Sample size N = 50. Figure S2. Mean consumption (g/day/individual ± SEM) (a) and survival rate ± SEM (b) of O. asiaticus feeding on three plant species as measured under laboratory conditions The horizontal axis indicates the plant species. Sample size, N = 60 The values of the bar chart are mean ± SEM. Error bar indicates the standard error. Those marked by different lowercase letters are significantly different based on Tukey’s HSD at P < 0 05. Figure S3. The partial least-square modelling path on grasshopper performance. Figure S4. The effect of plant biomass composition on grasshopper body size from partial least-square modelling (Red arrows indicate negative effects, blue arrows indicate positive effects). [file 12898_2019_248_MOESM1_ESM.pdf]

Additional Materials

Additional Tables

Table S1. The factors that are used for orthogonal design

Level / Factors	1	2	3
A *S. krylovii* (g)	20	40	60
B *L. chinensis* (g)	20	40	60
C *A. frigida* (g)	20	40	60

The three factors refer to the three plants. A: *Stipa krylovii*. B: *Leymus chinensis*. C: *Artemisia frigida*. The three levels (1, 2, 3) of fresh plant biomass are 20, 40, and 60 g.

Table S2. Orthogonal design and their factors

Factors / EXPNO	A	B	C	Block
1	1	1	1	1
2	1	2	2	2
3	1	3	3	3
4	2	1	2	3
5	2	2	3	1
6	2	3	1	2
7	3	1	3	2
8	3	2	1	3
9	3	3	2	1

L_9 (3^4) orthogonal table. A, B, and C refer to the three plant factors. A: *Stipa krylovii*. B: *Leymus chinensis*. C: *Artemisia frigida*. The numbers in the columns indicate the factor levels, 1 mean low level, 2 mean intermediate level, and 3 mean high level.

Table S3. Variance analysis of the orthogonal test for mortalities of *O. asiaticus* from third instar to adults within manipulated plant community compositions

**Source**	**df**	**ANOVA SS**	**Mean square**	***F* value**	**Pr > *F***
Block	1	0.0613	0.0613	5.13	0.0470
S. krylovii	2	0.0211	0.01056	0.88	0.4432
L. chinensis	2	0.0119	0.0060	0.50	0.6209
A. frigida	2	0.0486	0.0243	2.03	0.1814
Model	7	0.1429	0.0204	1.71	0.2131
Error	10	0.1194	0.0119		
Corrected total	17	0.2624			

Table S4. Partial least-square path modelling of the effects (coefficient) of plant biomass, food availability and quality, and grasshopper population density on grasshopper body size

Relationships	direct	indirect	total	P value
Plant biomass -> Food availability and quality	-0.0496	0	-0.0496	0.907
Plant biomass -> Density	-0.8107	0.027	-0.7837	**0.00179**
Plant biomass -> Grasshopper body size	0.979	-0.351	0.6282	0.1487
Food availability and quality -> Density	-0.5447	0	-0.5447	**0.00974**
Food availability and quality -> Grasshopper body size	0.8829	-0.213	0.6695	0.0887
Density -> Grasshopper body size	0.3918	0	0.3918	0.5701

Additional Figures

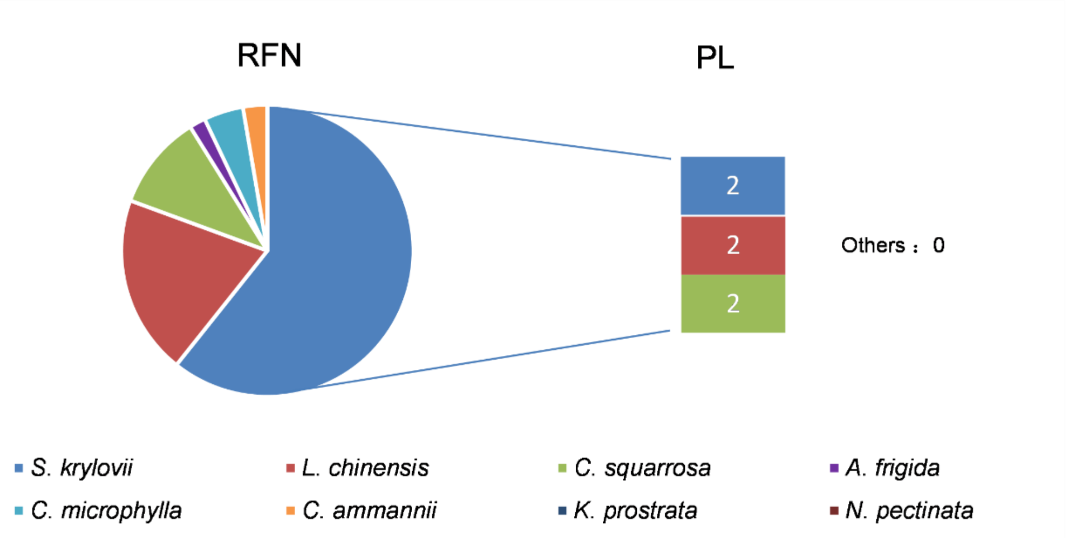

Figure S1 Feeding frequency and preference level of eight plants *PL*s for *S krylovii*, *L chinensis*, *C squarrosa* are 2 Other plants are defined as 0 PL: preference level; 0: not consumed (<0 5% of the plant eaten); 1: limited consumption with 0 5–30% of the plant eaten; 2: high consumption with > 30% of the plant eaten Sample size $N = 50$

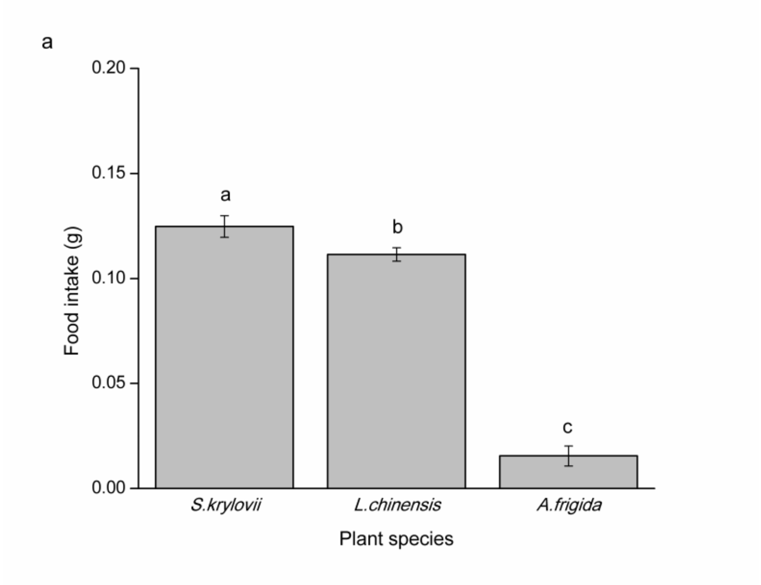

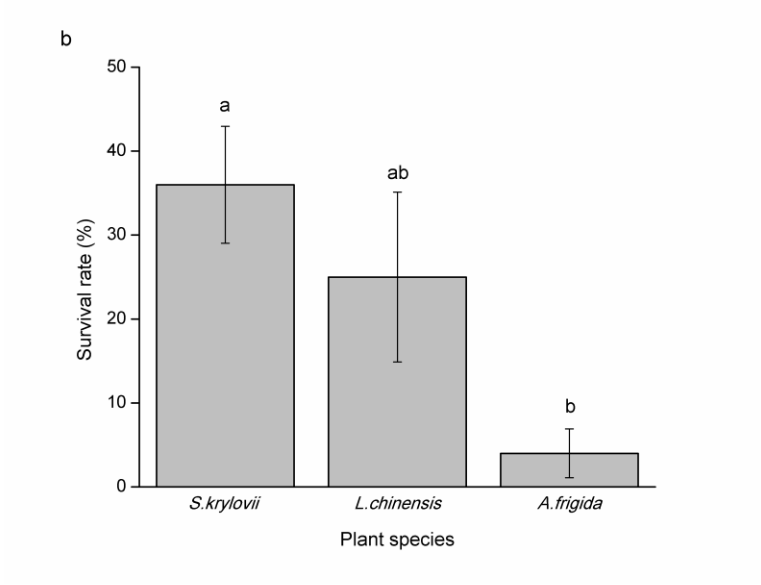

Figure S2 Mean consumption (g/day/individual ± SEM) (a) and survival rate ± SEM (b) of *O asiaticus* feeding on three plant species as measured under laboratory conditions The horizontal axis indicates the plant species Sample size, N =60 The values of the bar chart are mean ± SEM Error bar indicates the standard error Those marked by different lowercase letters are significantly different based on Tukey's HSD at P < 0 05

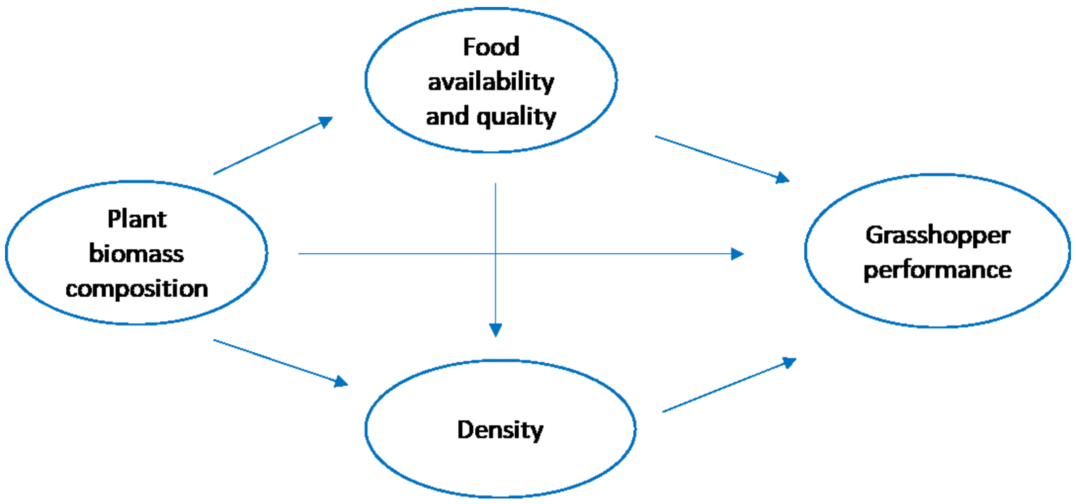

Figure S3. The partial least -square modelling path on grasshopper performance.

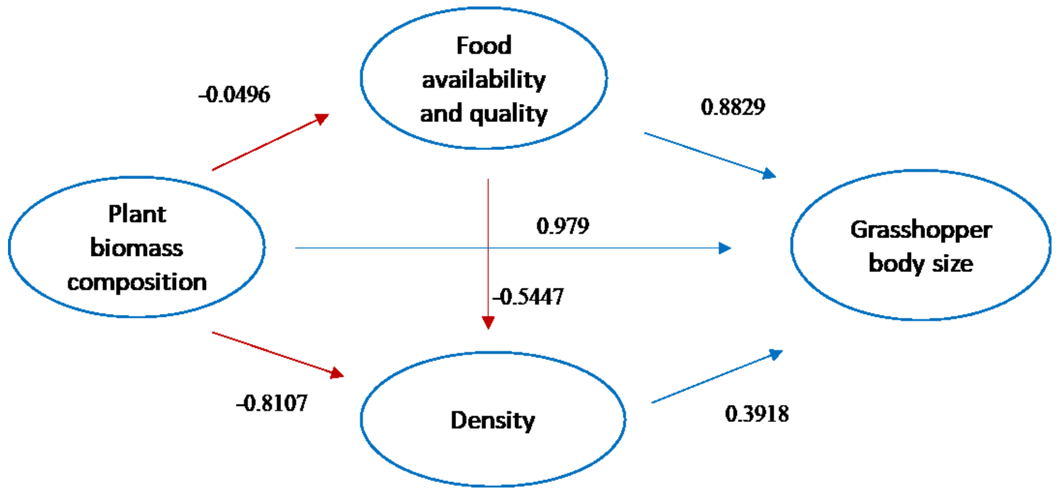

Figure S4. The effect of plant biomass composition on grasshopper body size from partial least - square modelling.
